# Adeno-Associated Virus Monoinfection Induces a DNA Damage Response and DNA Repair That Contributes to Viral DNA Replication

**DOI:** 10.1128/mbio.03528-22

**Published:** 2023-01-31

**Authors:** Kang Ning, Cagla Aksu Kuz, Fang Cheng, Zehua Feng, Ziying Yan, Jianming Qiu

**Affiliations:** a Department of Microbiology, Molecular Genetics and Immunology, University of Kansas Medical Center, Kansas City, Kansas, USA; b Department of Anatomy and Cell Biology, University of Iowa, Iowa City, Iowa, USA; Indiana University Bloomington

**Keywords:** AAV, monoinfection, DDR, DNA repair, DNA replication, DNA damage response, adeno-associated virus

## Abstract

Adeno-associated virus (AAV) belongs to the *Dependoparvovirus* genus of the *Parvoviridae* family. AAV replication relies on a helper virus, such as adenovirus (Ad). Co-infection of AAV and Ad induces a DNA damage response (DDR), although its function in AAV DNA replication remains unknown. In this study, monoinfection of AAV2 in HEK293T cells expressing a minimal set of Ad helper genes was used to investigate the role of the DDR solely induced by AAV. We found that AAV2 DNA replication, but not single stranded (ss)DNA genome accumulation and Rep expression only, induced a robust DDR in HEK293T cells. The induced DDR featured the phosphorylation of replication protein A32 (RPA32), histone variant H2AX (H2A histone family member X), and all 3 phosphatidylinositol 3-kinase-related kinases (PIKKs). We also found that the kinase ataxia telangiectasia and Rad3-related protein (ATR) plays a major role in AAV2 DNA replication and that Y family DNA repair DNA polymerases η (Pol η) and Pol κ contribute to AAV2 DNA replication both *in vitro* and in HEK293T cells. Knockout of *Pol η* and *Pol κ* in HEK293T cells significantly decreased wild-type AAV2 replication and recombinant AAV2 production. Thus, our study has proven that AAV2 DNA replication induces a DDR, which in turn initiates a DNA repairing process that partially contributes to the viral genome amplification in HEK293T cells.

## INTRODUCTION

Adeno-associated parvoviruses (AAVs) are a group of nonenveloped T = 1 icosahedral viruses, belonging to the genus *Dependoparvovirus* in the family *Parvoviridae* (1). AAV contains a single stranded (ss)DNA genome of ~4.7 kb, flanked by 2 identical inverted terminal repeats (ITRs) that form palindromic structures. Group A of AAVs expresses 2 large (Rep78/68) and 2 small (Rep52/40) nonstructural proteins, and group B of AAVs, e.g., AAV5, expresses only Rep78 and Rep52 (2, 3). In addition, 2 auxiliary nonstructural proteins, assembly-activating protein (AAP), and membrane-associated accessory protein (MAAP) have been identified during AAV infection or recombinant (r)AAV production (4–6). All AAVs express 3 viral structural proteins (VP1, VP2, and VP3) for the assembly of a capsid at a ratio of 1:1:10 (7). Rep78/68 proteins, containing a DNA replication origin binding/endonuclease domain, a helicase domain, and a transcription activation domain, are essential for AAV DNA replication (8). Rep52/40 proteins facilitate the packaging of the synthesized ssDNA genome into capsids (9). MAAP protein is postulated to limit AAV production and promote AAV egress (5, 6), and AAP facilitates capsid assembly (4, 10–12).

AAV DNA replicates through a so-called DNA strand displacement mechanism (13, 14). Using the hairpin-like ITR as a primer, the ssDNA viral genome is converted into a duplex replicative intermediate. Then, Rep78/68 bind to the Rep-binding sites at the ITR region, unwind the duplex genome, and nick ssDNA strand at the terminal resolution site (*trs*), which leads to the synthesis of a cDNA strand. At the end, the folding of an ITR from the unwinding of the duplex DNA initiates DNA synthesis through strand displacement. Replication factor C (RFC), replication protein A (RPA), proliferating cell nuclear antigen (PCNA), DNA polymerase δ, and the minichromosome maintenance (MCM) complex are the essential host factors for AAV DNA replication, by which AAV2 DNA replication can be reconstituted *in vitro* in test tubes (15–18).

As a dependoparvovirus, AAV relies on a helper virus, such as adenovirus (Ad) (19), herpes simplex virus (20), vaccinia virus (21), human papillomavirus (22), or human bocavirus 1 (HBoV1) (23), to complete its replication. Expression of a minimal set of Ad helper genes (*E1*, *E2a*, *E4orf6*, and *VAI RNA*) is sufficient to support AAV DNA replication in the nucleus where Rep and capsid (Cap) proteins accumulate and progeny virus production (24, 25). However, helper-independent AAV replication can occur, generating infectious viral progenies. Exogenous DNA damage induced by UV irradiation is fully supportive for a productive AAV infection in the absence of a helper virus (26). Importantly, AAV2 and Ad coinfection induced a robust DNA damage response (DDR) that was distinct from the response induced by Ad alone and largely dependent on AAV2 DNA replication (27, 28). However, the role of the AAV-induced DDR during AAV infection remains elusive, and more specifically, how the induced DDR potentially contributes to viral DNA replication is unknown.

DDR is involved in the replication of other parvoviruses. In the productive infection of the autonomous parvoviruses, minute virus of mice (MVM) interacted with cellular DNA damage foci (29), and the large nonstructural NS1 protein facilitated the localization of viral genome to the DNA damage foci (30). HBoV1 infection initiates a DDR with activation of all 3 phosphatidylinositol 3-kinase–related kinases (PI3KKs), including ATM (ataxia telangiectasia mutated), ATR (ATM and RAD3 related), and DNA-PKcs (DNA-dependent protein kinase catalytic subunit) (31, 32). Importantly, 2 members of the Y family DNA repair DNA polymerases, polymerase η (Pol η) and Pol κ, are recruited to the viral DNA replication centers and play an important role in viral DNA replication (31, 32). Notably, MVM infects dividing cells, and HBoV1 replication occurs in the nondividing, well-differentiated human airway epithelium (32, 33). While AAV infects both dividing and nondividing cells (23, 34), how AAV DNA replicates in post-mitotic cells where the DNA replication machinery is in quiescence remains largely unknown.

In this study, with the helper functions provided through co-transfection of a minimal set of Ad helper genes, we used AAV2 productive monoinfection to study AAV2 DNA replication and the induced DDR in HEK293T cells. We confirmed that AAV2 DNA replication, but not the viral genome accumulation or Rep expression only, induced a robust DDR in HEK293T cells. The DDR was featured by hallmarks of phosphorylation of RPA32, histone variant H2AX (H2A histone family member X), and all 3 PI3KKs. The role of DNA damage and repair in AAV2 DNA replication was validated by both *in vivo* in cells and *in vitro* viral DNA replication assays in test tubes. We also provided evidence that the DNA repair DNA polymerases, Pol η and Pol κ, play important roles in AAV2 DNA replication.

## RESULTS

### AAV2 DNA replication, but not viral proteins, induces a robust DDR.

**FIG 1:**
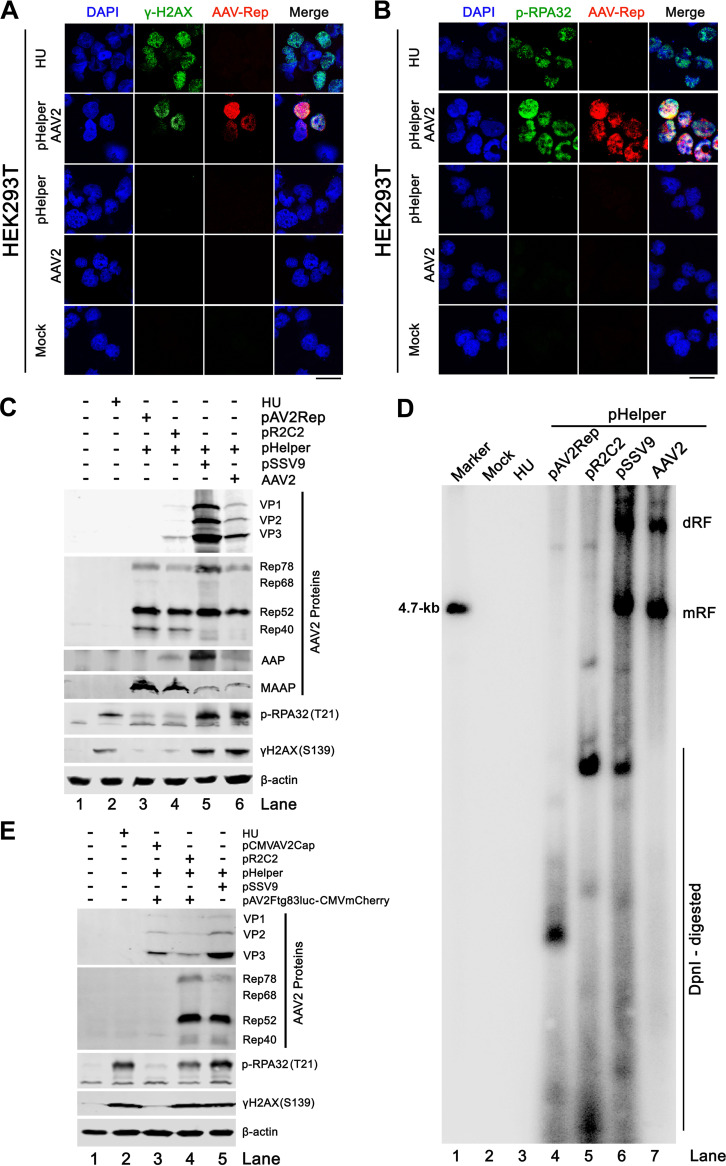
AAV2 monoinfection in HEK293T cells induces a DDR. (A and B) HEK293T cells were infected with AAV2 alone or transfected with pHelper and infected with AAV2 or mock-infected, as indicated. At 2 dpi, the cells were collected for immunofluorescence assays using antibodies against the indicated proteins. (C to E) HEK293T cells were transfected with the plasmids as indicated. At 2 days postransduction (dpt), cell lysates were prepared for Western blotting with antibodies against the indicated proteins (C and E), and Hirt DNA samples were prepared for Southern blotting (D) with an AAV2 *repcap* probe. Ten ng of AAV2 duplex DNA of PvuII-digested SSV9 was used as a marker (M). HU-treated cells served as DDR positive control, and β-actin served as a loading control. Bar = 25 μm.

Coinfection of AAV2 with Ad5 induces a DDR (27, 28). While Ad infection alone induces a DDR (27), whether or how the replication of AAV2 itself induces a DDR is still obscure. To address this question, we investigated AAV2 infection in HEK293T cells with the helper functions provided by pHelper plasmid transfection, instead of Ad infection. In this case, HEK293T cells were transfected with the pHelper plasmid that expresses Ad2 E2a, E4, and VA RNA (35), followed by AAV2 infection. At 2 days postinfection (dpi), immunofluorescence staining showed 2 DDR hallmarks: phosphorylated RPA32 at threonine (T)21 (p-RPA32) and phosphorylated H2AX at serine (S)139 (γH2AX) in the AAV2 Rep-expressing (infected) cells (Fig. 1A and B, pHelper/AAV2). Notably, transfection of pHelper alone did not induce expression of p-RPA32 and γH2AX (Fig. 1A and B, pHelper). In the absence of pHelper transfection, neither expression of p-RPA32 and γH2AX nor Rep expression were detected in AAV2 infected cells (Fig. 1A and B, AAV2). Western blotting further confirmed the 2 DDR hallmarks in AAV2 infected cells transfected with pHelper (Fig. 1C, lane 6).

AAV2 Rep78/68 expression was the only AAV component that has been reported to induce a mild DDR (28, 36). Whether the newly discovered AAV nonstructural proteins, AAP and MAAP, induce a DDR is unknown. To this end, HEK293T cells were co-transfected with pHelper and an AAV2 ITR-deleted plasmid (pR2C2) that expresses all AAV2 proteins, including Rep, Cap, AAP, and MAAP (Fig. 1C, lane 4): an AAV2 Rep-expressing plasmid pAV2Rep that expresses all Rep proteins and MAAP (Fig. 1C, lane 3), and an AAV2 infectious clone (pSSV9) (Fig. 1C, lane 5), respectively. While transfection of pR2C2 and pAV2Rep induced minimal expression of p-RPA32 and γH2AX, pSSV9 transfection and AAV2 infection induced stronger p-RPA32 and γH2AX expression (Fig. 1C, lanes 3 and 4 versus 5 and 6). MAAP expression alone did not induce p-RPA32 and γH2AX expression (data not shown). AAV2 infection and pSSV9 transfection in pHelper transfected HEK293T cells showed active viral DNA replication, but not pR2C2 and pAV2Rep transfections (Fig. 1D, lanes 4 and 5 versus 6 and 7).

**FIG 2:**
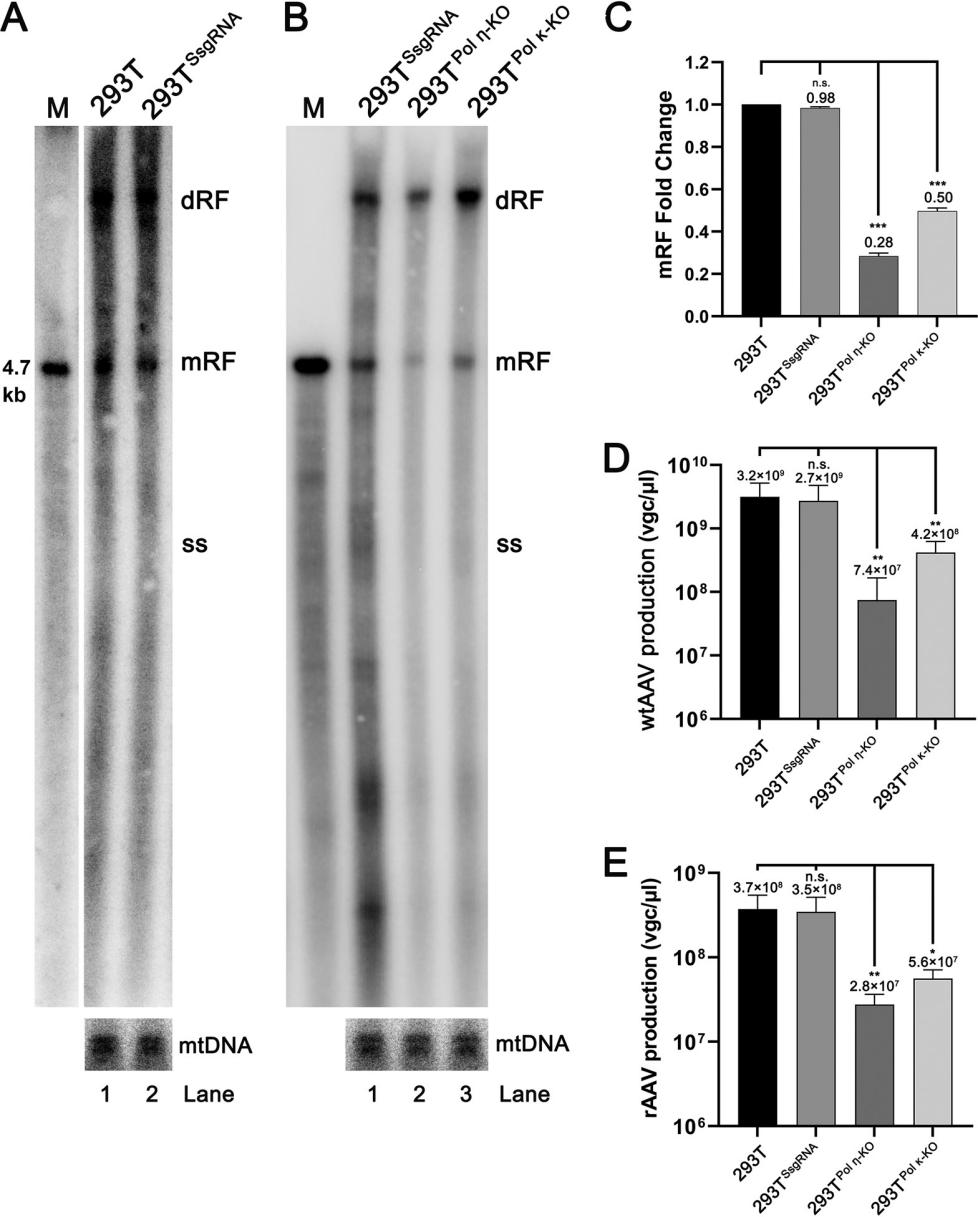
Knockout of DNA repair DNA polymerases significantly reduces AAV2 replication. (A and B) Southern blotting. HEK293T and HEK293T^SsgRNA^ (A) and HEK293T^Pol η-KO^, HEK293T^Pol κ-KO^, and HEK293T^SsgRNA^ (B) cells were transfected with pHelper and infected with AAV2. At 2 dpi, Hirt DNA was extracted from the infected cells and analyzed using Southern blotting with the AAV2 *repcap* probe. mtDNA was probed as a loading control. Representative blots are shown. dRF, double replicative form; mRF, monomer replicative form; and ssDNA, single-stranded DNA. AAV2 DNA recovered from PvuII-digested pSSV9 was used as a size maker (M). Probed mtDNA served as a loading control. (C) Quantification of mRF DNA. The bands of mRF DNA, normalized to the amount of mtDNA, were quantified. Relative fold changes are shown with means and standard deviations calculated from 3 independent experiments, which were normalized to the amount of mtDNA. (D) Quantification of wtAAV2 virus production. One 150-mm plate of HEK293T, HEK293T^SsgRNA^, HEK293T^Pol η-KO^, and HEK293T^Pol κ-KO^ cells were transfected with pHelper and infected with AAV2. At 2 dpi, the cells were harvested for wtAAV2 preparation. The virus resuspended in 500 μL were quantified for the production of progeny virus. The average numbers of virus genome copies (vgc) of the progeny virions per preparation and standard deviations are shown. (E) Quantification of rAAV production. One 150-mm plate of HEK293T, HEK293T^SsgRNA^, HEK293T^Pol η-KO^, and HEK293T^Pol κ-KO^ cells were co-transfected with pHeIper, pR2C2, and pAVF5tg83luc-CMVmChery. At 72 hpt, the cells were harvested for rAAV2 preparation in 500 μL. The vgcnumbers of wtAAV2 and rAAV2 per μL are shown with averages and standard deviations. *, *P* < 0.05; **, *P* < 0.01; ***, *P* < 0.001; n.s., no statistically significant difference.

To further confirm that the replication of AAV2 genome induces a DDR, we also examined whether a DDR was induced by the replication of rAAV2 genome when rAAV2 was produced in HEK293T cells. Thus, we transfected AAV2 transgene plasmid (pAV2Ftg83luc-CMVmCherry), pR2C2 (AAV2 Rep and Cap expression plasmid), and pHelper in HEK293T cells, which generated rAAV2 (Fig. 2E). The results showed that, like AAV2 replication, the production of rAAV2 in HEK293T cells induced p-RPA32 and γH2AX expression at levels as high as those in the SSV9 replication system (Fig. 1E, lane 4 versus 5). When the expression of Rep proteins was withdrawn from the transfection, p-RPA32 and γH2AX expression was remained at the background level in transfected HEK293T cells (Fig. 1E, lane 3 versus 1). In this case, the replacement of the pR2C2 with pCMVAV2Cap that only expresses AAV2 Cap proteins led to no replication of rAAV2 genomes.

Taken together, these results confirmed that the expression of AAV2 Rep proteins induces a weak DDR; however, active AAV2 DNA replication from either AAV2 monoinfection or transfection of replicable AAV2 DNA is necessary for a robust DDR.

### AAV2 monoinfection activates ATM, ATR, and DNA-PKcs, with ATR activation playing a major role in AAV2 DNA replication.

**FIG 3:**
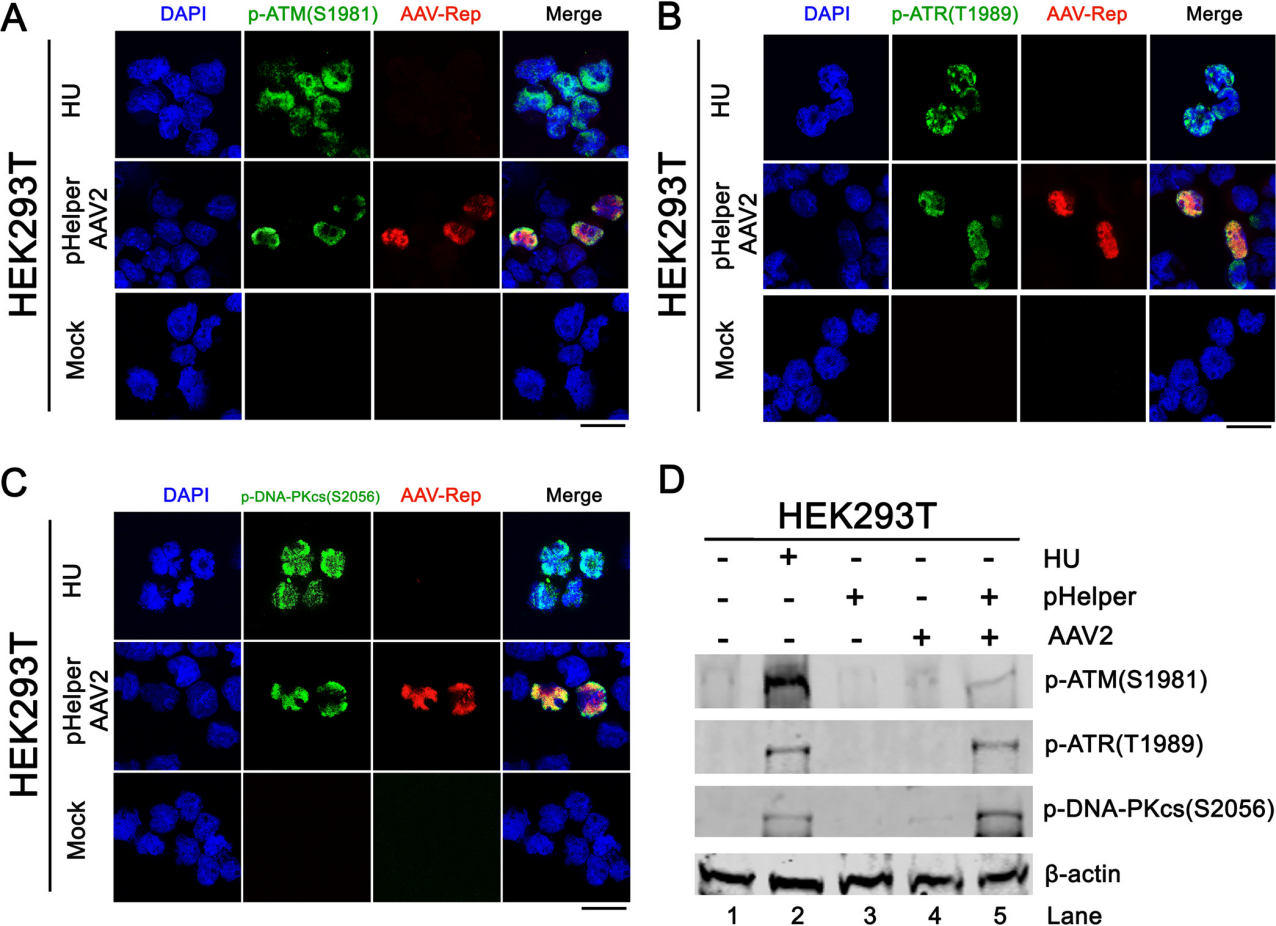
AAV2 monoinfection activates ATM, ATR, and DNA-PKcs. HEK293T cells were transfected with pHelper, followed by AAV2 infection or mock infection. At 2 dpi, cells were collected for immunofluorescence assays (A to C) and Western blotting (D), with specific antibodies against the proteins as indicated. HU-treated cells were used as a DDR positive control. Anti-phosphorylated (p)-ATM (S1981), anti-p-ATR (T1989), and anti-p-DNA-PKcs (S2056) antibodies were used for the detection of the phosphorylated forms of ATM, ATR, and DNA-PKcs proteins, respectively. β-actin served as a loading control. Bar = 25 μm.

We next examined the activation status of the three PI3KKs in AAV2 infected cells. Immunofluorescence assays showed that phosphorylated ATM at serine 1981 [p-ATM (S1981)] (37), ATR at threonine 1989 [p-ATR (S1989)] (38), and DNA-PKcs at serine 2056 [p-DNA-PKcs (S2056)] (39) in AAV2 infected cells were associated with Rep expression (Fig. 3A to C), suggesting the DNA damage foci marked with phosphorylated PI3KK colocalized with Rep-expressing foci, the viral DNA replication centers. Western blotting confirmed that ATM, ATR, and DNA-PKcs were phosphorylated (Fig. 3D). These results confirmed that AAV2 infection in HEK293T cells activated ATR, ATM, and DNA-PKcs.

**FIG 4:**
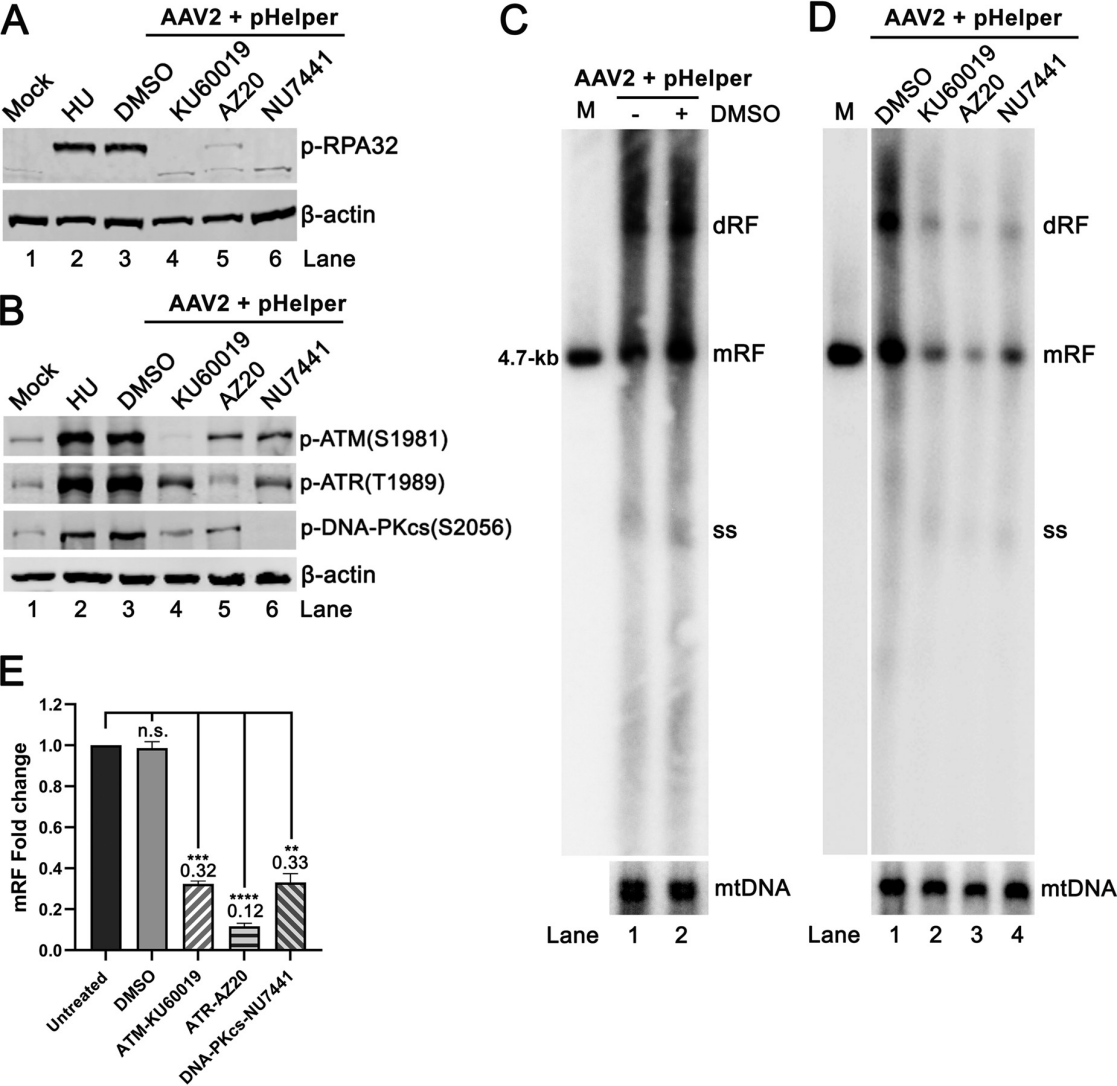
Treatment of pharmacological inhibitors that specifically target ATM, ATR, or DNA-PKcs impairs AAV2 replication in HEK293T cells. HEK293T cells were treated with vehicle control DMSO or pharmacological inhibitors targeting ATM (KU60019), ATR (AZ20), and DNA-PKcs (NU7441), respectively, at 2 days prior to pHelper transfection and AAV2 infection. (A and B) Western blotting. At 2 dpi, the cells were collected for Western blotting using specific antibodies targeting p-RPA32 (A) and p-ATM, p-ATR, and p-DNA-PKcs (B), respectively. HU-treated cells served as a DDR positive control. (C and D) Southern blotting. At 2 dpi, the cells were used to extract Hirt DNA for Southern blotting. The blot was probed for AAV2 DNA and mt DNA using an AAV2 *repcap* probe and a mtDNA probe, respectively. dRF, double replicative form; mRF, monomer replicative form; and ssDNA, single-stranded DNA. AAV2 DNA recovered from PvuII-digested pSSV9 was used as a size maker (M) at 4.7 kb. (E) Quantification of mRF DNA. The data were normalized to the amount of mtDNA and shown relative folds to the untreated cell group with means and standard deviations obtained from three independent experiments. *P* values were calculated using Student's *t* test (**, *P* < 0.01; ***, *P* < 0.001; ****, *P* < 0.0001; n.s., no statistically significant difference [*P* > 0.1]).

To further explore a role of PI3KK activation in AAV2 DNA replication, we first treated HEK293T cells with kinase-specific pharmacological inhibitors, prior to AAV2 infection and pHelper transfection. At 48 hours postinfection (hpi), the application of KU60019 (specific to ATM), AZ20 (specific to ATR), or NU7441 (specific to DNA-PKcs) efficiently inhibited the phosphorylation of RPA32 (Fig. 4A), and each inhibitor specifically inhibited the phosphorylation of its targeted kinase (Fig. 4B). Notably, as revealed by Southern blotting, while treatment of all the 3 pharmacological inhibitors significantly impaired AAV2 DNA replication, the ATR-specific inhibitor AZ20 decreased viral DNA replication the most by 88% by quantification of the monomeric replicative form (mRF) DNA (Fig. 4D and E). The treatment of dimethyl sulfoxide (DMSO) was used as a vehicle control and showed a negligible effect on viral DNA replication (Fig. 4C). The application of the 3 pharmacological inhibitors showed negligible effects on cell cycle progression (Fig. S1).

10.1128/mbio.03528-22.1 FIG S1 Treatment of pharmacological inhibitors targeting ATM, ATR, and DNA-PKcs does not alter cell cycle progression. (A) Cell cycle analysis. Pharmacological inhibitors target ATM (KU60019), ATR (AZ20), and DNA-PKcs (NU7441), respectively, were applied to HEK293T cells. At 2 days post-treatment, a BrdU incorporation assay was used to track *de novo* synthesized DNA. The cell cycle progression was analyzed using flow cytometry. (B) Quantification and statistical analysis. Different values represent percentages of the cell population in the indicated cell phases. Data represent means and standard deviations from 3 repeated experiments. n.s, no statistical significance. Download FIG S1, TIF file, 1.1 MB. Copyright © 2023 Ning et al. 2023 Ning et al. https://creativecommons.org/licenses/by/4.0/ This content is distributed under the terms of the Creative Commons Attribution 4.0 International license.

**FIG 5:**
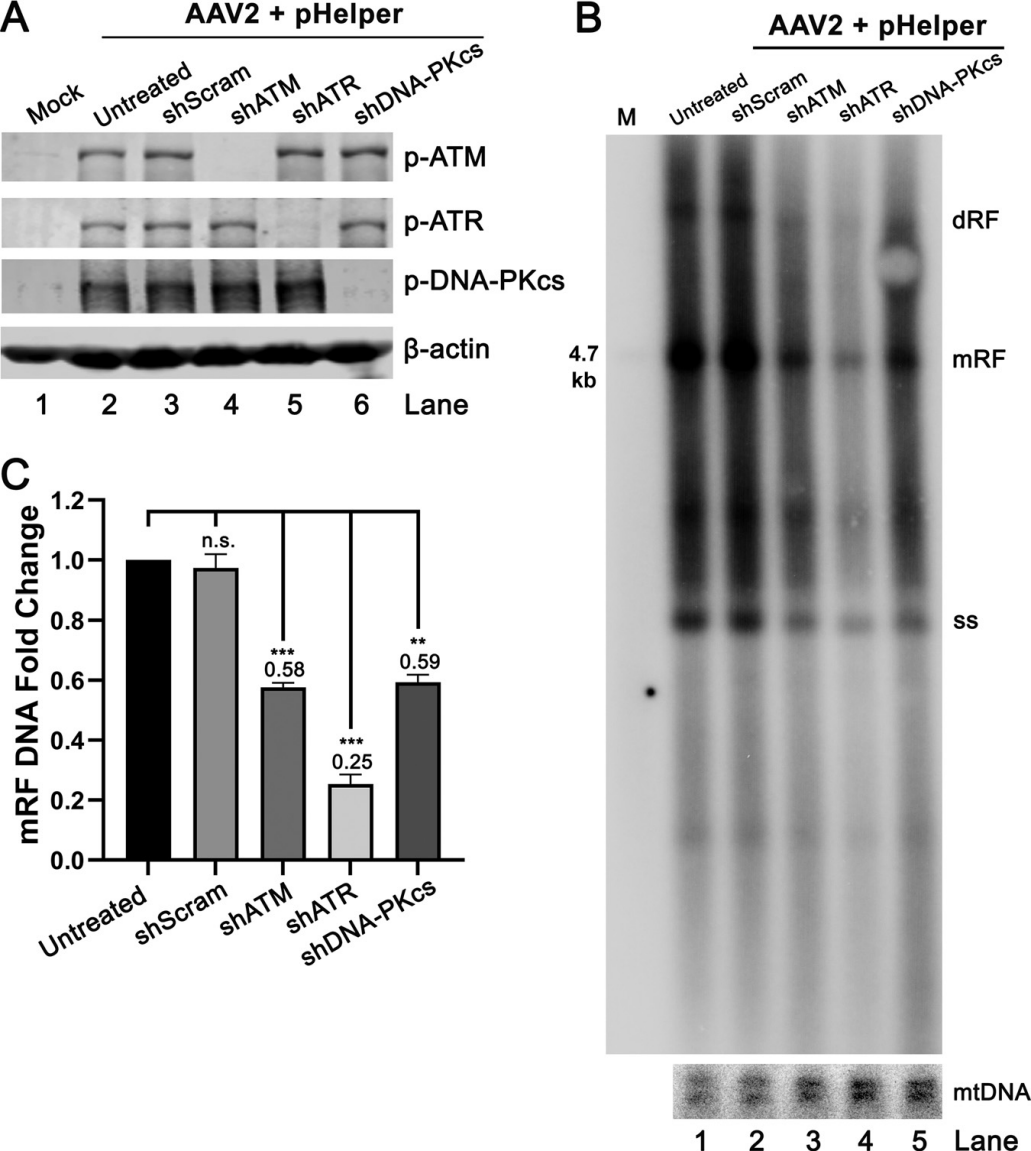
Knockdown of ATM, ATR, and DNA-PKcs decreases AAV2 replication. HEK293T cells were transduced with lentiviral vectors expressing kinase-targeted shRNA or scramble shRNA, as indicated, prior to pHelper transfection and AAV2 infection. (A) Western blot analysis. At 2 dpi, the cells were collected and lysed for Western blotting using anti-p-ATM(S1981), anti-p-ATR(T1989), and anti-p-DNA-PKcs(S2056) antibodies, respectively. β-actin was used as a loading control. (B and C) Southern blotting. At 2 dpi, the cells were used to extract Hirt DNA for Southern blotting. The blot was probed with an AAV2 *repcap* probe and a mtDNA probe, respectively. (B) A representative blot is shown. dRF, double replicative form; mRF, monomer replicative form; and ssDNA, single-stranded DNA. AAV2 DNA recovered from PvuII-digested pSSV9 was used as a size maker (M) at 4.7 kb. Probed mtDNA served as a loading control. (C) Quantification of mRF DNA. The data were normalized to the amount of mtDNA and represented on the results of 3 independent experiments. *P* values were calculated by using Student's *t* test (**, *P* < 0.01; ***, *P* < 0.001; n.s., no statistically significant difference).

We next employed a small hairpin RNA shRNA-mediated gene silencing strategy to knock down the expression of ATM, ATR, and DNA-PKcs, respectively, in HEK293T cells prior to AAV2 infection and pHelper transfection. To this end, the shRNA-expressing lentiviruses that previously succeeded in the knockdown of these genes (32) were used to transduce HEK293T cells. As expected, the transduction of the lentiviruses efficiently knocked down the expression of ATM, ATR, and DNA-PKcs, compared with the scramble shRNA control (shScram) (Fig. 5A). Southern blot analysis showed that the replication of AAV2 DNA in the ATM-, ATR-, and DNA-PKcs-knockdown cells were significantly decreased. Notably, the knockdown of ATR resulted in the largest reduction (by 75%) of AAV2 DNA replication, compared with the shScram transduced cells and the untreated cells (Fig. 5B and C, lane 4). This was consistent with the observation that the treatment of ATR inhibitor AZ20 was the most effective (by 88%) in reducing AAV DNA replication (Fig. 4D and E).

Collectively, these results confirmed that activation of ATM, ATR, and DNA-PKcs contributed to AAV2 DNA replication in HEK293T cells, among which the ATR activation played a major role.

### AAV2 infection does not induce cell cycle arrest in HEK293T cells.

**FIG 6:**
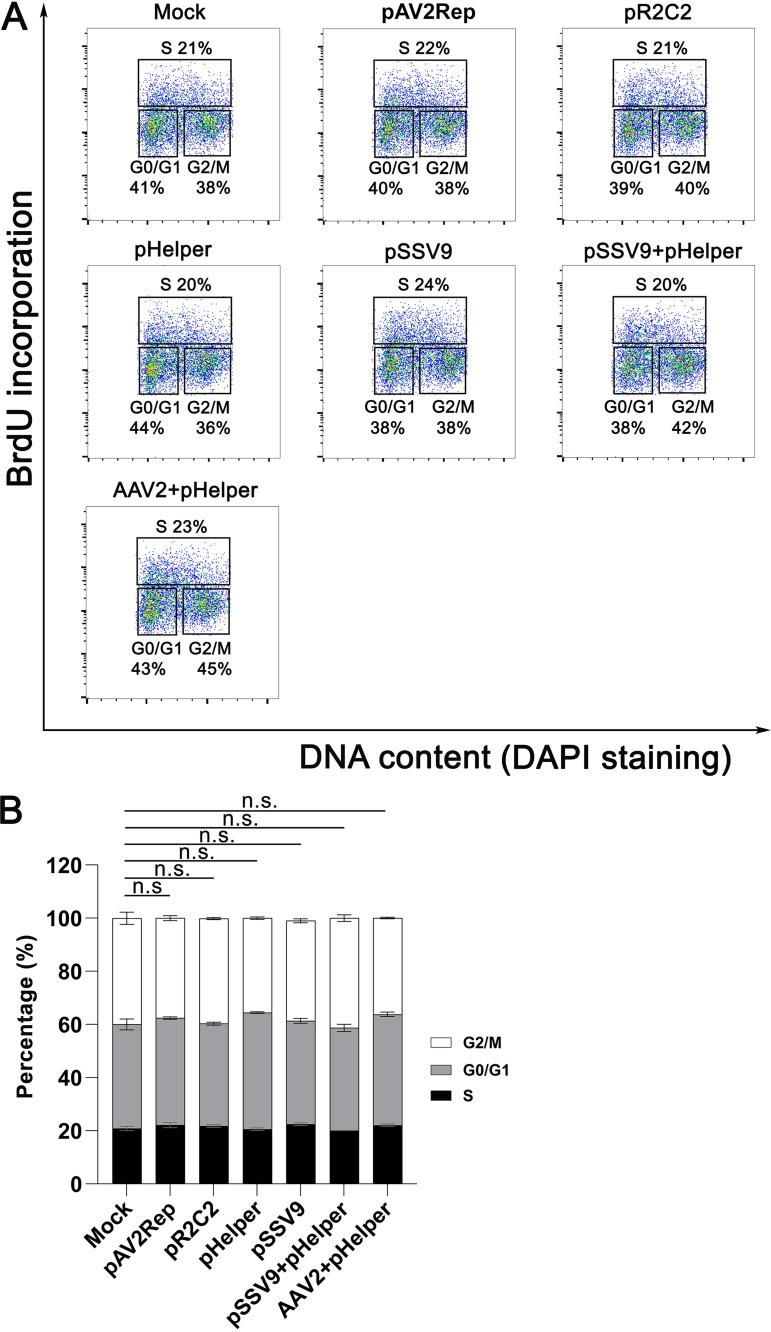
Neither transfection of AAV2 protein expressing plasmids or an infectious clone nor AAV2 infection induces cell cycle arrest. HEK293T cells were transfected with AAV2 plasmids as indicated or infected with AAV2. At 2 dpt/dpi, BrdU incorporation assay was used to track *de novo* synthesized DNA. The cell cycle progression was analyzed using flow cytometry. (A) Flow cytometry. A representative histogram of flow cytometry is shown with the percentages of the cells in G1, S, and G2/M phases of the sorted populations, as indicated. (B) Statistical analysis. The cell population at G0/G1, S, and G2/M phases, respectively, are shown with means and standard deviations calculated from 3 repeated experiments. n.s., no statistical significance.

As parvovirus infection induced DDR often arrests the cell cycle (40, 41), we examined the cell cycle alternation induced by expression of AAV2 proteins, replication of AAV2 DNA by the infectious clone pSSV9, and AAV2 infection, respectively. The results showed neither transfection of AAV2 Rep (pAV2Rep) or both Rep and Cap expressing plasmid (pR2C2), nor transfection of replicating pSSV9 plasmid with co-infection of pHelper induced cell cycle arrest (Fig. 6). Moreover, AAV2 infection of pHelper transfected HEK293T cells did not alter the cell cycle, compared with the cells only transfected pHelper (Fig. 6). These results strongly suggest neither AAV2 proteins nor AAV2 DNA replication induces cell cycle arrest of HEK293T cells.

### DNA repair DNA polymerases play an important role in wild-type AAV2 DNA replication and recombinant AAV production.

Y family DNA repair DNA polymerase Pol η and Pol κ have been demonstrated to have high fidelity during the DNA repair process (42), and importantly, they have been proven to participate in HBoV1 DNA replication (31, 32). We thus established single-cell-derived clonal HEK293T cell lines that have *Pol η* and *Pol κ* gene knockout (KO), respectively, using CRISPR-Cas9 genome editing technology. Genomic DNA sequencing confirmed *Pol η* and *Pol κ* KO (Fig. S2A and B), and Western blotting confirmed there was no expression of Pol η and Pol κ in the corresponding cell lines, when compared with the parent HEK293T and the Cas9/scramble single guide (Ssg)RNA-edited control cells (Fig. S2C). All the 3 lines of the CRISPR-edited cells (*Pol η*-KO, *Pol κ-*KO, and scramble single guide RNA [SsgRNA] control) proliferated similarly as the parent HEK293T cells. The *Pol η*-KO and *Pol κ-*KO cells were infected with AAV2 and transfected with pHelper. At 2 dpi, the cells were lysed, and the lower molecular weight (Hirt) DNA was extracted for Southern blotting (Fig. 2A and B). Quantification of monomeric replicated form (mRF) DNA in wtAAV2 infected cells showed a significant decrease of 72% and 50% in *Pol η* and *Pol κ* KO cells, respectively, compared with the SsgRNA control HEK293T^SsgRNA^ cells (Fig. 2C). The yield of progeny viruses from these cell lines were also evaluated. There were decreases in virus replication by 40 and 7 times in HEK293T^Pol η-KO^ and HEK293T^Pol κ-KO^ cells, respectively, compared with the control cells (HEK293T^SsgRNA^ and HEK293T) (Fig. 2D).

10.1128/mbio.03528-22.2 FIG S2 *Pol η* and *Pol κ* gene KO in HEK293T cells. (A and B) Sequences of the gRNA targeting region of the *Pol η* and *Pol κ* genes. 1 × 10^6^ cells of HEK293T^Pol η-KO^ and HEK293T^Pol κ-KO^ were collected. Genomic DNA was extracted using DNeasy blood and tissue kit (#69504, Qiagen). Primers complementary to upstream and downstream regions of the sgRNA targeting sequences were designed to amplify the indel region. Purified PCR DNA fragments were subjected to Sanger DNA sequencing at MCLAB (South San Francisco, CA). Representative results of sequencing chromatograms are shown. *Pol η* was disrupted at *Pol η* sgRNA targeted site (A), and *Pol κ* was disrupted at *Pol κ* sgRNA targeted site (B). Inserted nucleotides in the sgRNA targeted regions are indicated. (C) Western blot analysis of Pol η and Pol κ proteins. Lysates of the HEK293KT^Pol η-KO^, HEK293KT^Pol κ-KO^, and HEK293KT^SsgRNA^ were analyzed by Western blotting using anti-Pol η and anti-Pol κ specific antibodies, respectively. SsgRNA-expressing HEK293KT^SsgRNA^ cells served as control. β-actin was detected as a loading control. Download FIG S2, TIF file, 2.9 MB. Copyright © 2023 Ning et al. 2023 Ning et al. https://creativecommons.org/licenses/by/4.0/ This content is distributed under the terms of the Creative Commons Attribution 4.0 International license.

To further explore if Pol η and Pol κ are involved in rAAV production, the KO cell lines were transfected with pHelper, pR2C2, and pAV2F5tg83luc-mCherry to produce rAAV2. Following vector production, the results showed that the yield of rAAV2 decreased by 13- and 6-fold from HEK293T^Pol η-KO^ and HEK293T^Pol κ-KO^ cells, respectively, compared with the control cells (HEK293T^SsgRNA^ and HEK293T) (Fig. 2E).

With these results, we concluded that both DNA repair Pol η and Pol κ play a critical role in wtAAV2 replication and rAAV2 production, with Pol η having a more important role.

### Both DNA replication DNA polymerase and DNA repair DNA polymerases play a role in AAV2 DNA replication *in vitro*.

**FIG 7:**
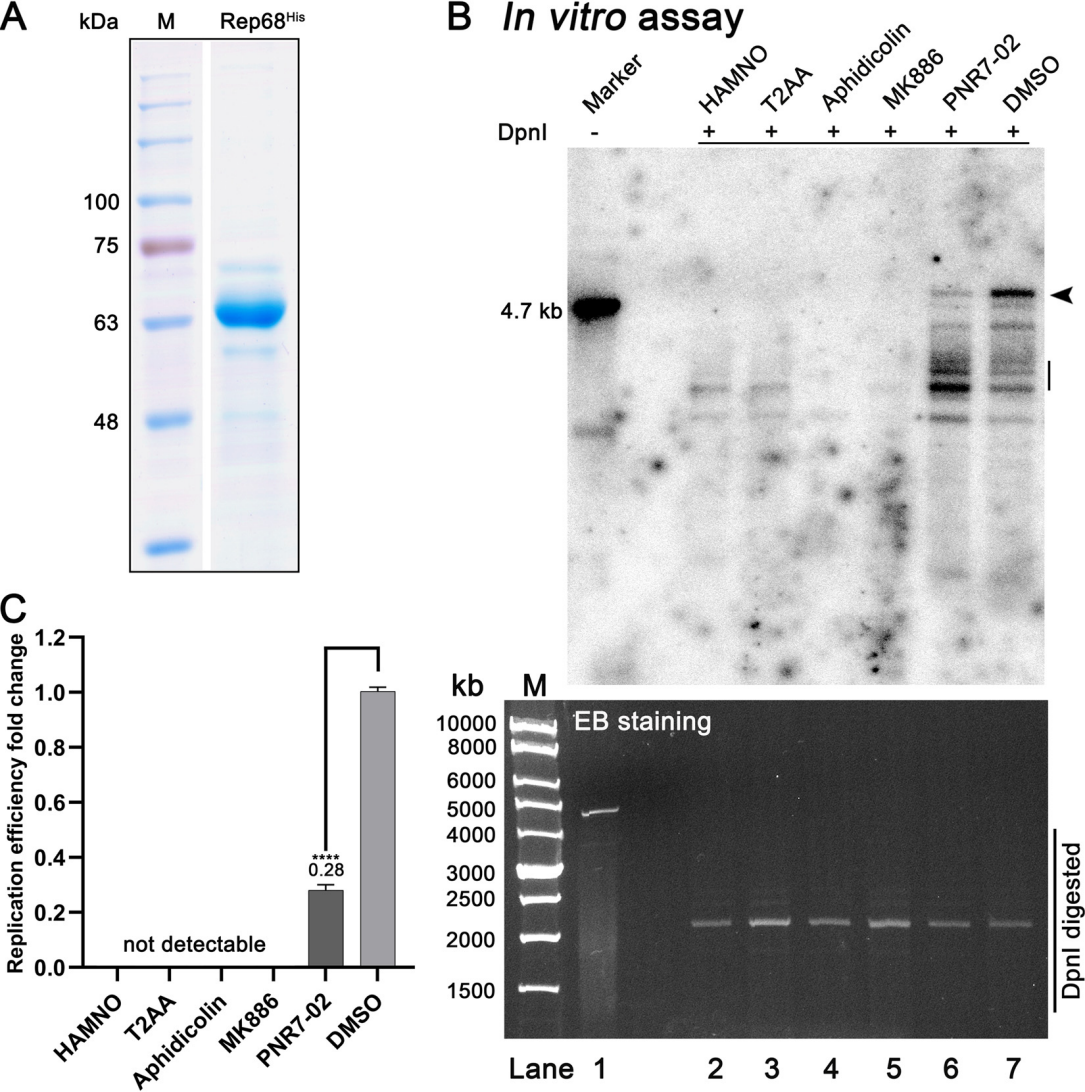
Pharmacological inhibitors targeting both DNA replication DNA polymerase and DNA repair polymerase abolish or significantly reduce AAV2 DNA replication *in vitro*. (A) Purification of AAV2 Rep68^His^ protein. AAV2 Rep68^His^ protein was purified as described in Materials and Methods. The eluted protein from peaked fractions were separated on an SDS-(12%) PAGE gel, along with a protein size ladder (M), and stained with Coomassie brilliant blue. (B) *In vitro* DNA replication assay. Various inhibitors HAMNO (specifically targeting to RPA complex) at 100 μM, T2AA (to PCNA complex) at 1 μM, aphidicolin (to Pol α/δ/ε) at 10 μg/mL, MK886 (to Pol κ/η) at 200 μM, and PNR7-02 (to Pol η/λ) at 20 μM were added into the reaction of *in vitro* replication assays. DMSO served as a vehicle control. After DpnI digestion for 1 h at 37°C, the *in vitro* replicated products (lanes 2 to 7) were resolved on 1% agarose gel, and stained with ethidium bromide (EB) for DpnI digested inputs (lower panel). The dehydrated gel was exposed to a phosphor screen and scanned on a Typhoon FLA 9000 scanner. (B, lane 1), the reaction product without DpnI digestion was loaded as a size maker (~4.7 kb). The arrowhead indicates the *in vitro* replicated AAV2 genome. (C) Quantification of the relative replication efficiency. The fully *in vitro* replicated (DpnI-digestion resistant) viral DNA signals at ~4.7 kb were quantified by using ImageQuant Tl (IQTL) 8.2 software (Cytiva). The quantities are presented as relative levels to the DMSO control (B, lane 7). The data were obtained from triple experiments. ****, *P* < 0.0001.

It has been reported that the RPA, PCNA, and MCM complexes along with Pol δ are essential factors for AAV2 DNA replication in HeLa cells (15, 16). They are also essential factors during eukaryotic cellular DNA replication (43). Thus, we evaluated the function of the cellular DNA replication machinery in AAV2 DNA replication with the *in vitro* replication assays using HEK293 cytosol extract supplemented with purified AAV2 Rep68 (Fig. 7A). In these assays, the addition of specific pharmacological inhibitors targeting RPA (HAMNO) (44), PCNA (T2AA) (45), and Pol α/δ/ε (Aphidicolin) (46) abolished the replication of the AAV2 full-length duplex genome (Fig. 7B, lanes 2 to 4), demonstrating the important role of the cellular DNA replication factors, PCNA and Pol δ, during AAV2 DNA replication. Notably, the RPA complex is required for both DNA replication and DNA repair processes (Fig. 7B, lane 2, and Fig. 7C). While the Pol η/κ-specific inhibitor MK886 (47) nearly abolished viral DNA replication (Fig. 7B, lane 5, and Fig. 7C), the addition of the Pol η-specific inhibitor PNR7-02 (48) decreased viral DNA replication to 28% when compared with the DMSO control reaction (Fig. 7B, lane 6 versus 7, and Fig. 7C). However, treatment of PNR7-02 resulted in a relative high level of incompletely replicated DNA that appeared at sizes smaller than the full-length duplex viral DNA (4.7 kb) (Fig. 7B, lane 6), suggesting that Pol η may play a role in repairing viral DNA replicated by Pol δ, which warrants further investigation.

Taken together, we confirmed that, along with cellular DNA replication factors, PCNA and DNA replication DNA polymerases (Pol δ) and Y family DNA repair DNA polymerases (Pol η/κ) play an important role in AAV2 DNA replication *in vitro*.

### AAV2 replication in other cell types induces negligible DDR.

**FIG 8:**
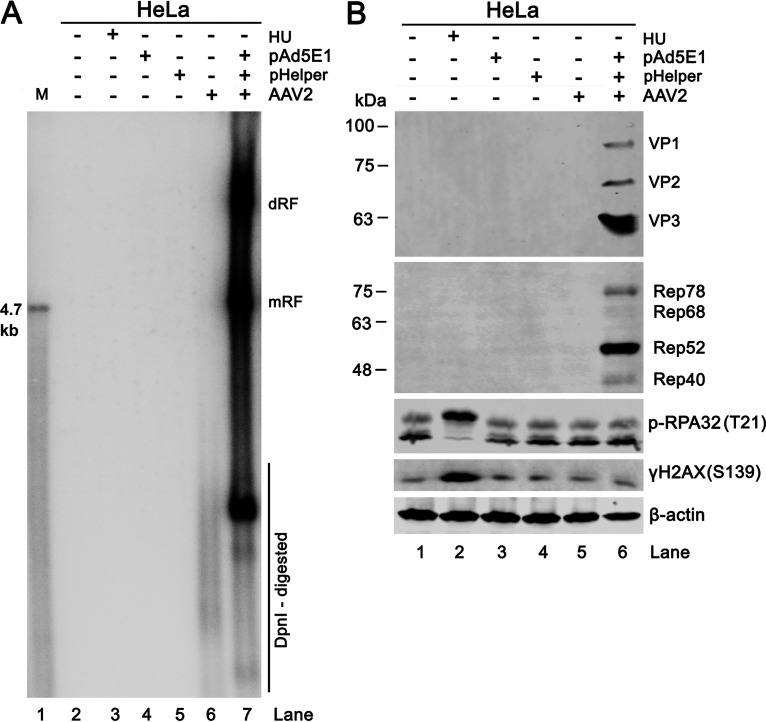
AAV2 replication in HeLa cells does not trigger a DDR. HeLa cells were transfected with the indicated plasmids and infected with AAV2 at an MOI of 5,000 vgc/cell or mock-infected as indicated. (A) Southern blotting. At 2 dpt/dpi, the cells were used to extract Hirt DNA, which was digested with DpnI and subjected to electrophoresis in 1% agarose gel, followed by Southern blotting. The blot was probed with an AAV2 *repcap* probe. dRF, double replicative form; mRF, monomer replicative form. AAV2 DNA recovered from PvuII-digested pSSV9 was used as a size maker (M). HU-treated cells were used as a positive control of DDR. (B) Western blotting. At 2 days post-treatment, the cells were collected and lysed for Western blotting to detect AAV2 capsid proteins (VP1/VP2/VP3), Rep proteins (Rep78/68/52/40), phosphorylated (p)-RPA32(T21), and γH2AX(S139). Β-actin was probed as a loading control.

As HEK293T cells stably expresse Ad5 E1 genes and SV40 large T antigen, we extended the study of AAV2 replication induced DDR to HeLa (Fig. 8) and other cell types, including U2-OS, Huh7, and A549 cells (Fig. S3). The cells were co-transfected with 2 Ad helper plasmids, pAd5E1 (expressing Ad5 E1 gene) and pHelper, followed by AAV2 infection at an MOI of 5,000. At 2 dpi, cell lysates were analyzed for expression of AAV2 Cap proteins and DDR hallmarks. AAV2 DNA replicated robustly in HeLa, U2-OS, Huh7, and A549 cells with the co-transfection of the 2 helper plasmids, as shown by Southern blotting of both mRF and dRF replicative viral DNA (Fig. 8A, lane 7, and Fig. S3A). AAV2 Rep and Cap proteins were detectable as expected (Fig. 8B, lane 6 and Fig. S3B to D, lane 6). However, AAV2 replication in these cells did not result in an obviously increased expression of p-RPA32 and γH2AX, when compared with the group that AAV DNA replication did not occur (Fig. 8B, lane 7 versus 6, and Fig. S3B to D, lane 6 versus 5).

10.1128/mbio.03528-22.3 FIG S3 AAV2 replication in U2-OS, Huh7, and A549 cells transfected with pAd5E1 and pHelper does not induce a DDR. Huh7, A549 and U2-OS cells were transfected with pAd5E1 and pHelper, followed by AAV2 infection at an MOI of 5,000 or mock-infected. (A) Southern blotting. At 2 dpi, Hirt DNA was extracted from the infected cells. After DpnI-digestion, Hirt DNA samples were resolved in 1% agarose gel, transferred to a nitrocellulose membrane, and probed for AAV2 DNA using an AAV2 *repcap* probe. dRF, double replicative form; mRF, monomer replicative form; and ss, single-stranded DNA. Marker was the AAV2 DNA recovered from PvuII-digested pSSV9. (B to D) Western blotting. At 2 dpi, the infected Huh7 (B), A549 (C), and U2-OS (D) cells were collected and lysed for detection of AAV2 capsid proteins (VP1/VP2/VP3), phosphorylated-(p)-RPA32(T21), and β-actin (a loading control). Download FIG S3, TIF file, 1.6 MB. Copyright © 2023 Ning et al. 2023 Ning et al. https://creativecommons.org/licenses/by/4.0/ This content is distributed under the terms of the Creative Commons Attribution 4.0 International license.

Collectively, we provided evidence that AAV2 DNA replication in HeLa, U2-OS, Huh7, and A549 cells did not induce a DDR.

### AAV genome accumulation *per se* does not trigger a DDR.

**FIG 9:**
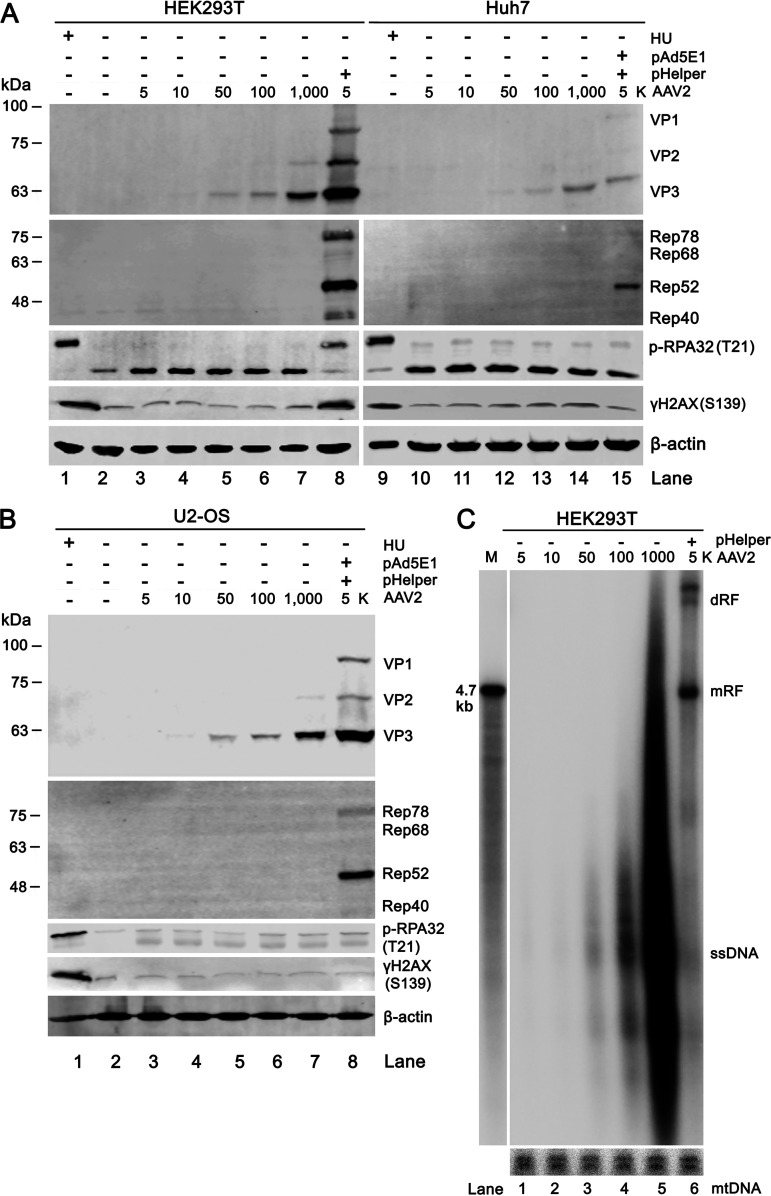
Accumulation of AAV2 genome in HEK293T, Huh7, and U2-OS cells does not induce a DDR. (A and B) Detection of p-PRA32 and AAV2 capsid proteins. HEK293T (A, left), Huh7 (A, right), and U2-OS (B) cells were infected with AAV2 at MOIs of 5,000 (5K), 10K, 50K, 100K, and 1,000K, respectively (A, lanes 3 to 7 and 10 to 14, and B, lanes 3 to 7). As an AAV2 replication control, cells were infected with AAV2 at an MOI of 5,000 and transfected with pHelper (A, lanes 8 and 15, and B, lane 8). At 2 dpi, the cells were collected and lysed for Western blotting. The blots were probed for AAV2 capsid proteins (VP1, VP2, and VP3), Rep proteins (Rep78/68/52/40), phosphorylated (p)-RPA32(T21), γH2AX(S139), and β-actin serving as a loading control. HU-treated cells were used as a DDR control. (C) Southern blotting of AAV2 genome in HEK293T cells. HEK293T cells were infected with AAV2 at an MOI of 5,000 (5K), 10K, 50K, 100K, and 1,000K, respectively. As an AAV2 replication control, cells were infected with AAV2 at an MOI of 5,000 and transfected with pHelper (C, lane 6). At 2 dpi, Hirt DNA was extracted from the infected cells, resolved in 1% agarose gel, and detected with a [α-32P]-dCTP-labeled AAV2 *repcap* probe and a mtDNA probe. dRF, double replicative form; mRF, monomer replicative form; ssDNA, single-stranded DNA; mtDNA as a loading control. AAV2 DNA recovered from PvuII-digested pSSV9 was used as a size maker (M).

Following AAV2 nuclear entry after infection, the ssDNA viral genome is uncoated in the nucleus and mimics a broken double-strand DNA, and might induce a DDR. It has been reported that AAV2 infection of U2-OS cells initiated partial viral DNA replication without the need of a helper virus or helper gene expression (49). To test the hypothesis that ssDNA viral genomes induce a DDR, HEK293T, Huh7, and U2-OS cells were infected with wtAAV2 at increasing MOIs (5K, 10K, 50K, and 100K to 1,000K) in the absence of pHelper plasmid transfection to prevent AAV2 gene expression and replication during these tests. AAV2 infected (MOI = 5K) and pHelper transfected cells served as a control for AAV2 replication. Notably, at 2 dpi, AAV2 Cap proteins were detected in a dose-dependent manner, but not AAV2 Rep proteins (Fig. 9A and B). Southern blot analysis did not detect any replicative form (RF) DNA in the cells infected with AAV2 only, compared with the AAV2 infection in the presence of Ad helper (Fig. 9C, lanes 1 to 5 versus 6). Thus, the detection of AAV2 Cap proteins were not due to AAV2 DNA replication, which likely came from the entered AAV2 in the cells. Notably, the DDR hallmark p-RPA32 was not detected in all the AAV2 infection only groups, including the infection at the highest MOI of 1,000K (Fig. 9A, lanes 2 to 7 and 10 to 14, and Fig. 9B, lanes 2 to 7).

Taken together, we confirmed that internalized AAV2 ssDNA genome in cells *per se* is not sufficient to induce a DDR even at a very high MOI (1,000K).

## DISCUSSION

In this study, we used the co-transfection of a minimal set of Ad helper genes and AAV2 monoinfection to study the AAV2 replication induced DDR, and a function of cellular DNA repair DNA polymerases in AAV2 DNA replication. DNA replication of AAV2 in HEK293T cells, but not in HeLa, Huh7, U2-OS, and A549 cells, induced a robust DDR with the phosphorylation of RPA32 and H2AX, as well as the activation of ATM, ATR, and DNA-PKcs. The induced DDR, in particular the ATR signaling, executes an important function in AAV2 genome amplification without an apparent cell cycle arrest. The Y family DNA repair DNA polymerases Pol η and Pol κ play a significant role in AAV2 genome amplification. Thus, our study provides evidence that replication of the AAV2 genome induces a robust DDR, and the recruited DNA repair machinery participates in AAV2 DNA replication and rAAV2 production in HEK293T cells.

### AAV2 DNA replicative intermediate accumulation mediates a DDR.

For autonomous parvoviruses, whose replication is independent on helper viruses, the large nonstructural protein (NS1) plays a critical role in the induction/initiation of the DDR. When only MVM NS1 was expressed in cells, NS1 colocalized with the DNA damage foci represented by γH2AX expression (30). During a MVM productive infection, NS1 bound to, and localized, the viral genome to cellular DNA damage foci to establish its replication (30). HBoV1 NS1 *per se* can initiate a DDR and activate ATM, ATR, and DNA-PKcs (31, 32), whereas the NS1 of parvovirus B19 (B19V) and minute virus of canines (MVC) did not (50, 51). For dependoparvoviruses, during coinfection with Ad, AAV2 replication triggers ATM and DNA-PKcs-dependent DDR pathways (27, 28). Although the Ku complex and DNA-PKcs interacted with Rep proteins (52, 53), the weak DDR induced by Rep expression implies that the large Rep protein was not accountable for all the DDR events during AAV2 replication in the presence of helper virus (28). In our study, we confirmed that expression of Rep proteins induced a mild DDR that was much weaker than that induced by AAV2 replication. The lines of evidence that ectopically expressed AAV2 Rep proteins colocalized with preexisting or induced cellular DNA damage foci, and that during AAV2 replication, both AAV2 genome and Rep localized to the cellular DNA damage foci (54), suggesting that Rep68/78 proteins interact with cellular DNA damage foci.

The single- and double-stranded DNA junctions of the AAV ITRs mimic the aberrant cellular DNA replication fork (55). It has been reported that infection of UV-inactivated AAV induced an ATR-dependent DDR (55, 56). However, subsequent work showed rAAV vectors were not sufficient to provoke a DDR (56). Consistently, we found that even at a high MOI (1,000K) (although AAV2 infection accumulated a high level of viral ssDNA genome in cells compared to UV-inactivated virus and rAAV infection), the ssDNA accumulation *per se* did not induce an increased expression of phosphorylated H2AX and/or RPA32, confirming that the viral genome alone is insufficient to induce a DDR. These lines of evidence suggest that the AAV2 DNA replication with accumulated replicative intermediates, but not the Rep or viral ssDNA genome, amplifies the DNA damage foci and re-localizes DNA damage sensors, kinases, and DNA repair factors to the foci. However, it is likely that Rep78/68 is first localized to the background DNA damage foci, and initiates a low level of DDR (54).

### AAV2 productive monoinfection only induces a DDR in HEK293T cells but not in other tested cells.

AAV2 nonproductive infection of U2-OS cells induced approximately one half (~45%) of the viral genomes localized to cellular DNA damage foci indicated by the presence of γH2AX (54). Infection of U2-OS cells with UV-inactivated AAV2 initiated a DDR that involved activation of ATR and Chk1 kinases (57). In both HeLa and 293 cells, AAV2 and Ad co-infection induces the phosphorylation of ATM, DNA-PKcs, RPA32, and H2AX (27, 28). However, our study demonstrated that, when sufficient Ad helper gene functions were provided in the context of plasmid transfection, AAV replication in HeLa, A549, Huh7, and U2-OS cells did not trigger a robust phosphorylation of H2AX and/or RPA32. These differences indicate that AAV2 productive monoinfection does not induce a DDR in HeLa, A549, Huh7, and U2-OS cells. Thus, unlike in HEK293T cells, DNA damage and repair processes are unlikely involved in AAV2 DNA replication in HeLa, A549, Huh7, and U2-OS cells.

### AAV2 productive monoinfection-induced DDR does not arrest cell cycle.

Parvoviruses contain ssDNA genomes, and utilize DDR to facilitate their genome replication (41). For autonomous parvoviruses, except for HBoV1 (31), the effect of DDR on viral DNA replication in dividing cells is mainly due to cell cycle arrest (41). MVM infection induces a DDR that is mainly mediated by ATM signaling, which arrests the cell cycle at G2/M, a pseudo-S phase (58). MVC replication in Walter Reed/3873D (WRD) canine cells and B19V replication in human primary erythroid progenitor cells induced a late S phase cell cycle arrest (40, 41). The prolonged late or pseudo-S phase enriches cellular DNA replication factors, such as RFC, PCNA, MCM, and DNA polymerase δ, and facilitates parvovirus DNA replication (59, 60). AAV2 Rep78 expression has only been reported to induce S phase cell cycle arrest of NIH3T3 and U2-OS cells (36, 61), which was not observed in AAV2 monoinfection or Rep expression by transfection in HEK293Tcells.

Therefore, the DDR-facilitated AAV2 DNA replication in HEK293T cells is not mediated by prolonging the S phase to accumulate the DNA replication factors for AAV DNA replication, which can explain the fact that AAV2 replicates in differentiated cells that are no longer capable of cell division (23, 34). Thus, AAV likely utilizes different DNA replication mechanisms in different types of cells.

### The ATR-mediated DNA repair machinery facilitates AAV2 DNA replication.

In productive monoinfection of AAV2, albeit the ATM and DNA-PK pathways play a significant role in AAV DNA replication, the ATR pathway plays the major role that demonstrated >75% reduction in viral DNA replication by silencing the *ATR* gene (Fig. 5B and C). The ATR pathway leads to activation of proteins, such as Rad9-Rad1-Hus1 (9-1-1) and Rad17-replication factor C 2 to 5 (Rad-RFC) complexes, and the accumulation of DNA repair factors into the DNA damage foci (62, 63). The RPA complex binds to ssDNA, and is a key signal to activate ATR (64, 65). Following DNA damage, RPA32 is hyperphosphorylated, which limits DNA replication but aids DNA repair by marking DNA damage foci for the recruitment of DNA repair factors (66–68), including the 9-1-1 complex and the Rad-RFC complex (65, 69). Importantly, RPA32 is implicated in the translesion DNA synthesis (TLS) DNA repair pathway (70), and RPA32 is phosphorylated and colocalized within the viral DNA replication centers of HBoV1 and AAV (28, 32).

HBoV1 infection in well-differentiated primary human airway epithelium supports AAV replication, where DNA replication factors, such as DNA polymerase δ, are absent (23). We proved here that DNA repair polymerases Pol η and Pol κ inhibitors significantly inhibited AAV replication *in vitro*, and more importantly, the knockout *Pol η* and *Pol κ* in HEK293T cells significantly decreased AAV2 replication and virus production of both wtAAV2 and rAAV, supporting the hypothesis that DNA repair processing is involved in AAV2 DNA replication in HEK293T cells. This DNA repair oriented AAV2 DNA replication should be the key for the AAV2 infection in well-differentiated cells. Pol η and Pol κ are Y family DNA repair DNA polymerases that have been reported to be involved in error-free DNA repair of TLS (42, 71, 72). TLS is responsible for TLS-assisted DNA lesion bypass outside the context of the replication fork when DNA damage results in ssDNA gaps (73). The ATR signaling induced 9-1-1 complex has a physical similarity to the homotrimeric sliding clamp PCNA (74). We propose that TLS DNA repair polymerases (Pol η and Pol κ) interact with the 9-1-1 complex, which locks the polymerases in a location that enables viral DNA synthesis. Thus, overexpression or induction of the ATR-mediated DNA repair machinery in HEK293T cells may increase rAAV production.

In summary, our study provides strong evidence that AAV utilizes different mechanisms to replicate viral DNA in different cell lines. Besides cellular DNA replication Pol δ, AAV also uses high fidelity DNA repair polymerases for viral DNA replication. Echoing the fact that HBoV1 and AAV replicate in differentiated cells (23), parvoviruses have evolved to hijack various cellular DNA synthesis mechanisms to facilitate amplification of their genome in different hosts.

## MATERIALS AND METHODS

### Cell lines.

**(i) Regular cell lines.** Human embryonic kidney (HEK)293T cells (#CRL-11268), HeLa cells (#CRM-CCL-2), and A549 cells (#CCL-185, ATCC) were obtained from ATCC, Manassas, VA. Huh7 (#JCRB0403, JCRB Cell Bank) and U2-OS (#HTB-96, ATCC) cells were kindly gifted from Drs. Ann L. Wozniak and Irfan Saadi at the University of Kansas Medical Center. The cells were cultured in Dulbecco’s modified Eagle’s medium (DMEM) (#SH30022, Cytiva Life Science) with addition of 10% fetal bovine serum ([FBS], #F0926, MilliporeSigma) at 37°C under 5% CO_2_ atmosphere. Suspension 293ES cells were purchased from Expression Systems (#94-007S), and cultured in ESF SFM Mammalian Cell Culture Medium (#98-001-01) as previously described (75).

**(ii) Gene knockout (KO) cell lines.** HEK293T cells were transduced with lentiCRISPRv2 expressing single guide (sg)RNAs targeting *Pol η* and *Pol κ* genes, respectively, or a SsgRNA. At 48 h postransduction, the transduced cells were selected with puromycin at 2 μg/mL. After 1 week of selection, the cells were plated in 96-well plates by limiting dilutions. When the single cell clone became confluent, the cells were expanded and screened for gene KO using Western blotting and Sanger sequencing (Fig. S2).

### Plasmid constructs.

**(i) AAV2 plasmids.** Plasmids pAV2Rep, pCMVAV2Cap, pAAVRep2Cap2 (pR2C2), and pAV2F5tg83luc-CMVmCherry (4.6-kb) have been described previously (35, 76). AAV2 infectious clone SSV9 (pSub201[-]), containing a full-length AAV2 genome (77), was a gift from Dr. R. J. Samulski at the University of North Carolina, Chapel Hill (78).

**(ii) Ad plasmids.** pAd5E1 cloned with the first 4,031 bp of the Ad5 genome (containing E1 gene) was purchased from OD260 Inc., Boise, ID (#QP-04/pAd1127). pHelper plasmid was purchased from Agilent Technologies, Inc., Santa Clara, CA (#240071).

**(iii) pLKO-shRNA constructs.** The shRNA-expressing constructs, pLKO-shScram, pLKO-shATM, pLKO-shATR, and pLKO-shDNA-PKcs have been previously described (31).

**(iv) pLentiCRISPRv2 constructs.** Three sgRNAs targeting *Pol η* were cloned in 3 plentiCRISPRv2-Pol η KO vectors (Pol η-1: 5′-GCA CAA GTT CGT GAG TCC CG-3′; Pol η-2: 5′-GAC TGA CCC ATG TGA AAC CA-3′; Pol η-3: 5′-CTG CTC CCA CGG TGA GCT GC-3′). The 3 sgRNAs targeting *Pol κ* in lentiCRISPRv2-Pol κ KO are Pol κ-1: 5′-TAG GTT CAA CAC ACC TGA CG-3′, Pol κ-2: 5′-ATC CAT GTC AAT GTG CAC TA-3′, and Pol κ-3: 5′-CTT CTC CTT TGT GCT ATC CA-3′. The lentiCRISPRv2-SsgRNA expresses a scramble sgRNA (SsgRNA), containing a random sequence of 5′-GTA TTA CTG ATA TTG GTG GG-3′.

### Plasmid DNA transfection.

HEK293T cells were transfected using PEI MAX (#24765-2; Polyscience, Inc.) as previously described (79). A total of 2 μg plasmid was used for each well of a 6-well plate, and 4 μg plasmid was transfected to a 60-mm dish, and a total of 30 μg plasmid was used for each 150-mm plate. HeLa, A549, Huh7, and U2-OS cells were transfected using Lipofectamine 3000 (#L3000015; ThermoFisher), following manufacturer’s instructions.

### Chemicals and treatments.

**(i) DNA damage inducer and PI3KK inhibitors.** Hydroxyurea (HU; #127-07-1; MilliporeSigma) was prepared as a stock solution at 200 mM, according to manufacturer’s instructions. PI3KK inhibitors, KU60019 (#4176; Tocris Bioscience), AZ20 (#S7050; Selleckchem), and NU7441 (#3712; Tocris Bioscience), were dissolved in DMSO at 10 mM. They were applied to cell cultures at 2 days prior to infection at the final concentrations, as we previously reported: HU at 5 mM, KU60019 at 5 μM, AZ20 at 3 μM, and NU7441 at 1 μM (31, 32, 80).

**(ii) *In vitro* DNA replication inhibitors.** We used HAMNO (#S0148, Selleckchem), T2AA (#21921, Cayman Chemical), aphidicolin (#14007, Cayman Chemical), MK886 (#1311, Tocris Bioscience), and PNR7-02 (#2965, Axon Medchem). Stock solutions were prepared in DMSO at 10 mM. The inhibitors were directly added into the reaction of *in vitro* replication assays at a final concentration of 100 μM for HAMNO, 1 μM for T2AA, 10 μg/μL for aphidicolin, 200 μM for MK886, and 20 μM for PNR7-02 as previously recommended (44–48).

### AAV production and quantification.

wtAAV2 was produced by infection of pHelper transfected HEK293T cells with AAV2 (#VR-680, ATCC). rAAV2 was produced in a 150-mm plate by transfection of HEK293T cells with pR2C2, pAV2F5tg83luc-CMVmChery, and pHelper plasmids using PEI Max. At 72 h posttransfection, the cells were collected, lysed, and treated with DNase I (#10104159001; MilliporeSigma), 10% Sodium deoxycholate (#D6750; MilliporeSigma), and 0.25% Trypsin-EDTA. The clarified cell lysates were subjected to cesium chloride density gradient ultracentrifugation, according to a previously described protocol (23, 35).

Titters of AAV2 and rAAV2 were quantified using quantitative real time (qPCR) with an amplicon and a TaqMan probe specifically targeting the AAV2 *rep* gene and *mCherry* gene, respectively, as DNase I-digestion resistant viral genome copies (vgc) per mL (23, 35).

### AAV monoinfection.

For AAV2 productive infection, HEK293T cells were transfected with pHelper, while HeLa, A549, Huh7, and U2OS cells were transfected with pAdE1 and pHelper. The cells were then infected with wtAAV2 at a multiplicity of infection (MOI) of 5,000 vgc/cell. In some cases, without helper function provided, AAV2 was directly applied to the recipient cells at MOIs as indicated in the relevant figures.

### Lentivirus production and transduction.

Lentiviruses were produced by transfection of HEK293T cells with the sgRNA-expressing plentiCRISPRv2 or shRNA-expressing pLKO plasmids, together with 2 packaging plasmids, psPAX2 and pMD2.G, using PEI MAX and concentrated through a 20% sucrose gradient by ultracentrifugation in a SureSpin 630 rotor (Thermo Scientific) at 19,400 rpm for 3 h. The transduction unit of the produced lentiviruses were titrated as previously described (81).

For lentivirus transduction, HEK293T cells were transduced at an MOI of ~5 transduction units/cell. At 2 days postransduction, the cells were treated with puromycin at a final concentration of 2 μg/mL to select the transduced cells or carry out single cell cloning.

### Cell cycle analysis.

We performed a bromodeoxyuridine (BrdU) incorporation assay for cell cycle analysis, as previously described (82). Briefly, BrdU (#B5002; MilliporeSigma), prepared as a stock solution in distilled H_2_O at 10 mM, was added to HEK293T cells at a final concentration of 30 μM. After incubation at 37°C for 30 min, the cells were collected, fixed in 2% paraformaldehyde (PFA), and permeabilized with 0.5% Triton X-100 for 10 min. After denaturing chromosome DNA with 1 M HCl and neutralization with Boric acid-NaOH (pH 8.0), the cells were incubated subsequentially with a mouse anti-BrdU, an Alexa Fluor 488-conjugated anti-mouse secondary antibody, and DAPI (4’,6-diamidino-2-phenylindole). The cells were analyzed on a LSRII Flow cytometer (BD Biosciences), and data were analyzed using FlowJo software (FlowJo, LLC).

### Immunofluorescence assay.

Immunofluorescence staining was performed as previously described (83). Briefly, cells were washed twice with PBS, cytospun onto slides at 1,800 rpm for 3 min, and fixed with 3.7% PFA at room temperature for 30 min. Following washing with PBS three times, the cells were permeabilized with 0.5% Triton X-100 for 5 min, incubated with a primary antibody in PBS with 2% FBS at 37°C for 1 h, and subsequently with a correspondent secondary antibody. Nuclei were stained with DAPI. Confocal images were captured under a Leica TCS SP8 STED 3x Super Resolution Microscope.

### Western blotting.

Cell lysates were prepared at 48 hpi and separated with 2.5 μL of a protein ladder (#P008; GoldBio) on sodium dodecyl-sulfate polyacrylamide gel electrophoresis (SDS-PAGE) gels (84). The separated proteins were transferred onto a polyvinylidene difluoride (PVDF) membrane (#IPVH00010; MilliporeSigma), and sequentially blocked with 5% nonfat milk and probed with primary and secondary antibodies. The specific signals were visualized by an Odyssey imaging system (LI-COR Corporate).

### Southern blotting.

Southern blotting was essentially performed according to our previously reported methods (85). Briefly, lower molecular weight (Hirt) DNA samples were prepared from cells as described (75), and resolved in 1% agarose gel. The separated DNAs were transferred onto a nitrocellulose membrane (#1212590; GVS North America) and probed with an [α-^32^P]-dCTP-labeled probe of the AAV2 *repcap* gene. In some experiments, the blot was additionally probed for mitochondrial DNA (mtDNA) using a specific probe (32). After overnight exposure with a storage phosphor screen, hybridization signals were captured and visualized on an Amersham Typhoon Biomolecular Imager (Cytiva), and quantified using ImageQuant Tl (IQTL) 8.2 (Cytiva).

### Protein expression and purification.

The coding sequence of AAV2 Rep68, together with a 6 × histidine codons at the C-terminus, was cloned into pET30a (+) (#69909, MilliporeSigma) via NdeI and XhoI restriction sites. The constructed plasmid was then transformed into BL21/DE3 pLysS Escherichia coli bacteria (#L1195; Promega) for the expression of Rep68 with a His tag, AAV2 Rep68^His^. The recombinant protein was purified using a Bio-ScaleTM Mini NuviaTM IMAC cartridge (#7800811; Bio-Rad) on a Bio-Rad NGC Chromatography System as previously described (75).

### *In vitro* viral DNA replication assay.

The *in vitro* viral DNA replication assay was performed as described previously (75). Briefly, the cytoplasmic extract (S100) of 293 cells was prepared from the suspension culture of 293E cells collected at log growth phase. The standard *in vitro* viral DNA replication reaction mixture (50 μL) contains: 5 μL of 10 × reaction buffer (40 mM HEPES-KOH, pH 7.5; 7 mM MgCl_2_; 0.5 mM DTT), 40 mM phosphocreatine di(tris) salt (pH 7.0) (#P1937, MilliporeSigma), 2 μg of creatine phosphokinase (#C3755, MilliporeSigma), 25 μCi of [α-32P]-dCTP (#BLU013Z250UC, Perkin Elmer), 2.5 μL of 20 × NTPs/dNTPs (4 mM ATP; 200 μM CTP/GTP/UTP; 100 μM dATP/dTTP/dGTP; 30 μM dCTP), 200 μg of S100 of 293ES, 200 ng of the AAV2 duplex genome from PvuII-digested pSSV9, and 2 μg of the purified AAV2 Rep68^His^ protein.

The reaction mixture was incubated at 37°C overnight. Then, we added 5 μL 10 × Stop buffer (5 mM EDTA, 0.5% [wt/vol]SDS) and digested it with 1 μL proteinase K (10 mg/mL; #P2308; MilliporeSigma) at 37°C for 1h. The samples were purified with the Oligo Clean & Concentrator Kit (#D4061; Zymo Research) and digested with DpnI (#R0176; NEB) to remove the input AAV2 bacterial DNA. The *in vitro* replicated viral DNA samples were then separated in 1% agarose gel and dried on a filter paper on a gel dryer (Heto Dry GD-1, Heto Lab Equipment). The autoradiography signals were captured by exposing the dried gel to a storage phosphor screen overnight, visualized on the Amersham Typhoon Biomolecular Imager (Cytiva), and quantified using ImageQuant Tl (IQTL) 8.2 (Cytiva).

### Antibodies used in the study.

**(i) First antibodies.** The following first antibodies were used: Mouse anti-phospho-H2AX (Ser139) (#05-636), mouse anti-β-actin (#A5441) from MilliporeSigma rabbit anti-phospho- RPA32 (Thr21) (#AP1040), rabbit anti-phospho-DNA-PKcs (Ser2056) (#AP0621) and, rabbit anti-Pol κ (#A6122) from Abclonal, rabbit anti-phospho-ATR (Thr1989) (#GTX128145) and rabbit anti-polymerase η (#GTX109938) from GeneTex, mouse anti-AAV2 Rep protein (#03-61069; #03-61073) and rabbit anti-AAV2 capsid proteins (#03-61084) from ARP. Rat anti-MAAP was generated by immunizing rats with purified GST-MAAP, following a previously published protocol (86). Rabbit anti-AAP antisera were made by immunization of 2 rabbits with a peptide antigen, Cys-RSTSSRTSSARRIKDASRR (87), at Biomatik Co.

**(ii) Secondary antibodies.** The following secondary antibodies were used: DyLight 800 conjugated anti-rabbit IgG (#5151S) and DyLight 800 conjugated anti-mouse IgG (#5257S) (Cell Signaling, DyLight 800 conjugated anti-rat IgG (#SA5-10024) ThermoFisher, FITC conjugated anti-mouse IgG (#115-095-003), FITC conjugated anti-rabbit IgG (#111-095-003), Alexa Fluor 488 conjugated goat anti-mouse IgG (#115-545-062), and Alexa Fluor 594 conjugated goat anti-mouse IgG (#115-585-146) (Jackson ImmunoResearch).

### Statistical analysis.

Statistical analysis was performed with GraphPad Prism 9 (GraphPad Software). Data is representative of triplet experiments. The data shown indicate means and standard deviations. *P* values of statistical significance were analyzed by using Student’s *t* test. ****, *P* < 0.0001; ***, *P* < 0.001; **, *P* < 0.01; and *, *P* < 0.05 were regarded as statistically significant, and n.s. represents no statistical significance.
