# Supplementary figures and images for: Adeno-Associated Virus Monoinfection Induces a DNA Damage Response and DNA Repair That Contributes to Viral DNA Replication
Source: mBio. 2023 Jan 31;14(1):e03528-22. doi: 10.1128/mbio.03528-22 (PMC9973366; doi:10.1128/mbio.03528-22)

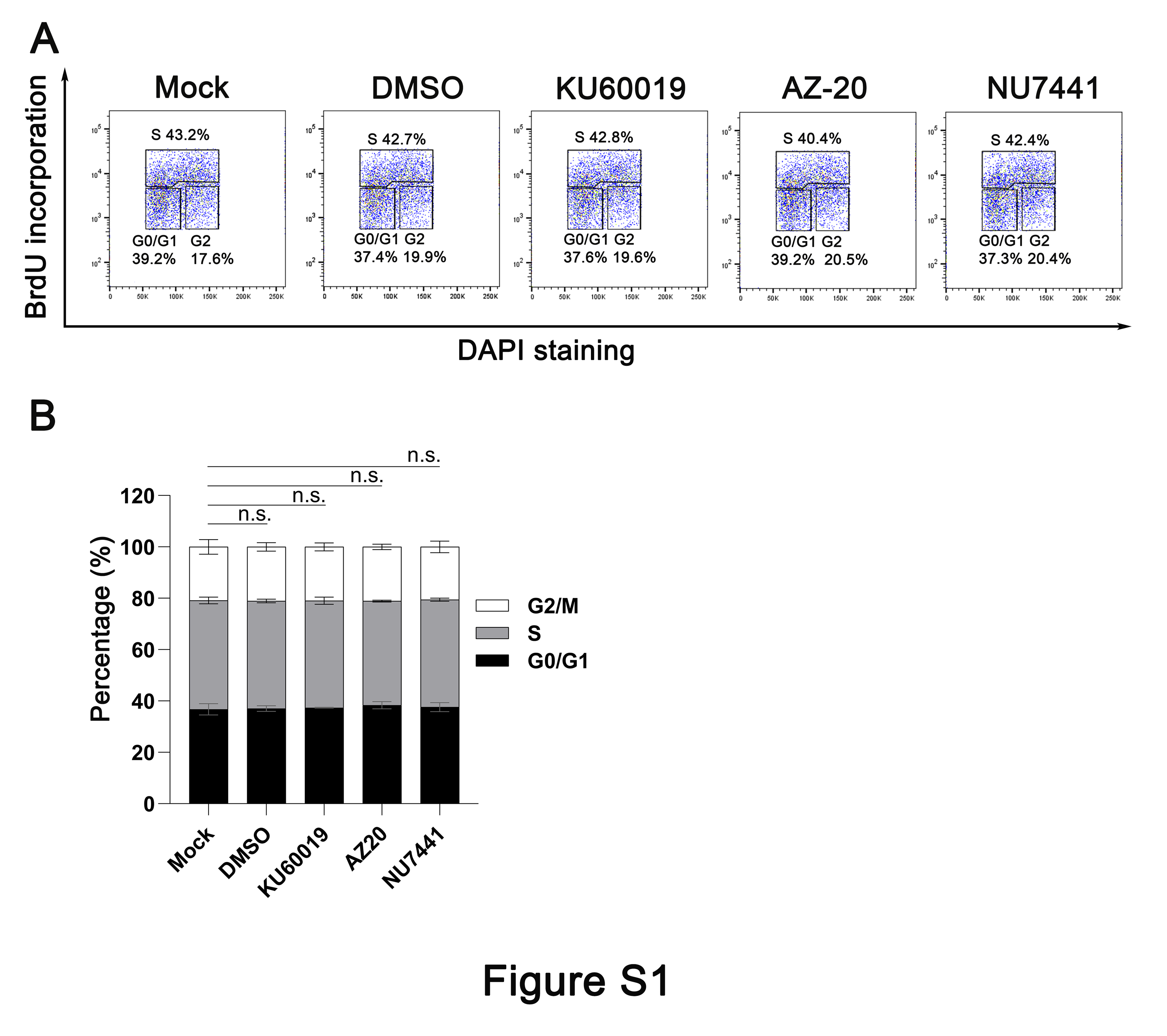

Supplement: FIG S1 [file mbio.03528-22-s0001.tif]

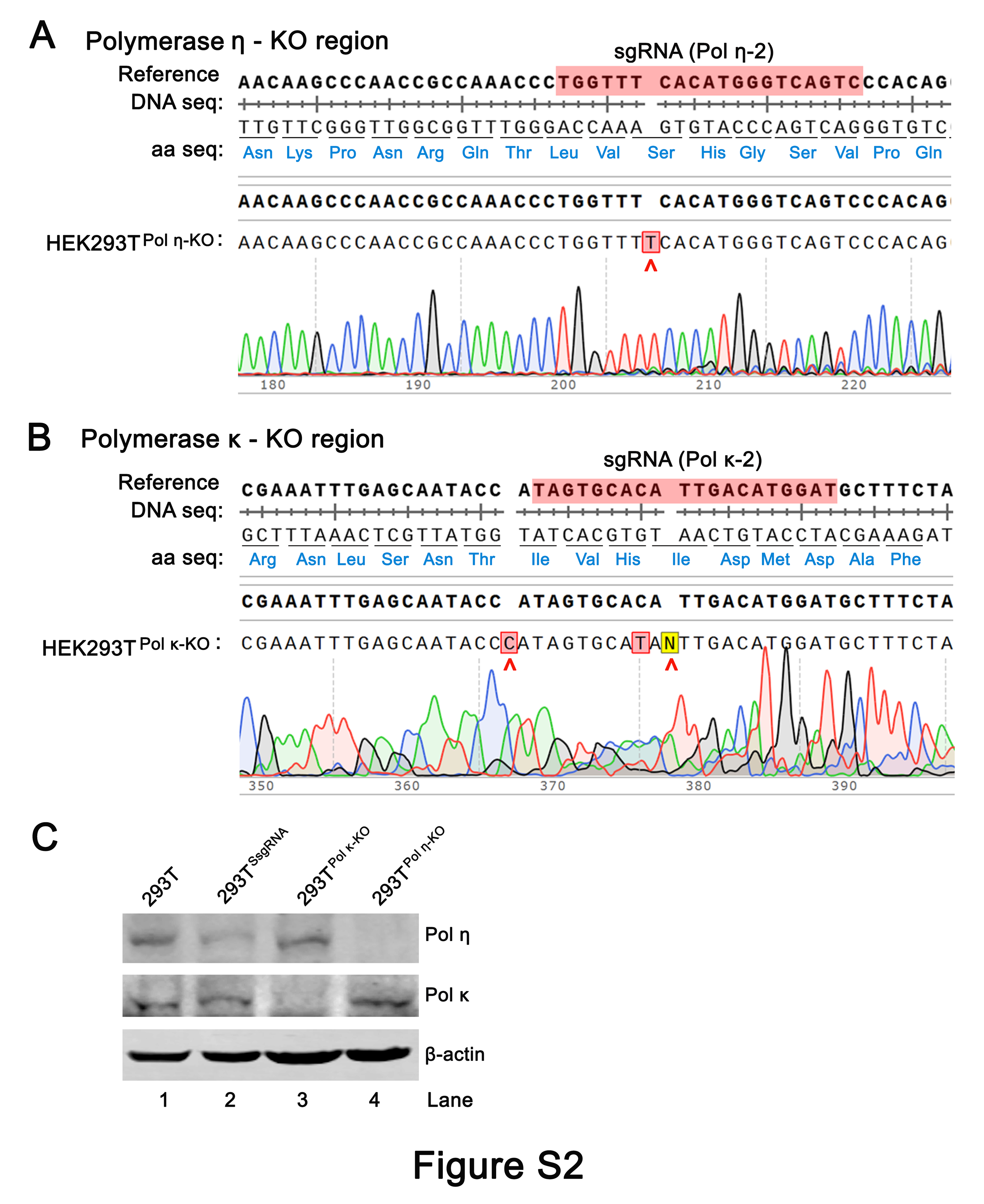

Supplement: FIG S2 [file mbio.03528-22-s0002.tif]

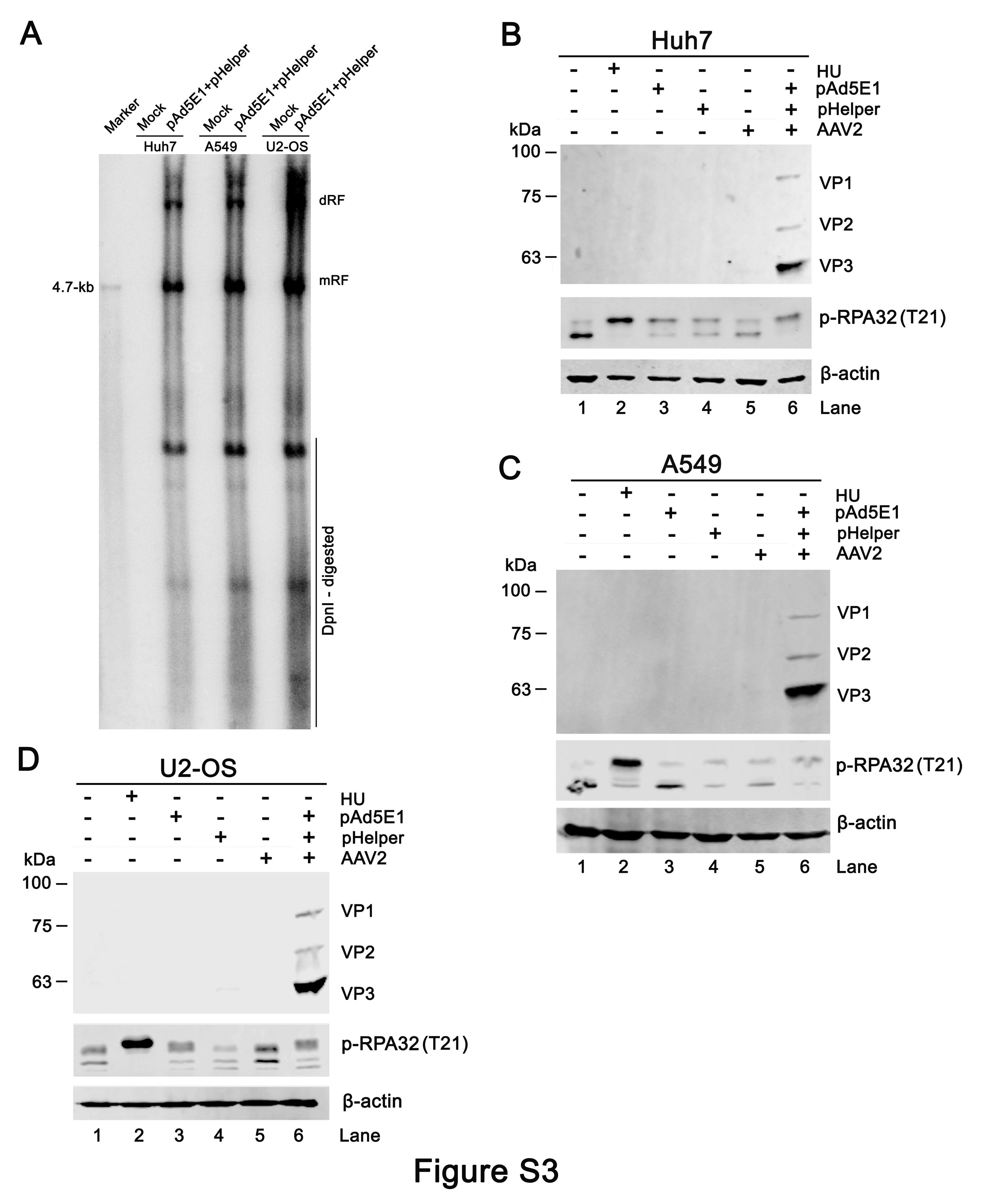

Supplement: FIG S3 [file mbio.03528-22-s0003.tif]
